# Comparison of Surface Roughness of Zirconia Polished with Novel Silicon Carbide Polishing Paste and Diamond Polishing Paste

**DOI:** 10.1055/s-0045-1812863

**Published:** 2025-11-11

**Authors:** Thanaphum Rattanadilok Na Phuket, Niyom Thamrongananskul, Atikom Surintanasarn, Suparaksa Yamockul

**Affiliations:** 1 Department of Esthetic Restorative and Implant Dentistry, Faculty of Dentistry, Chulalongkorn University, Bangkok, Thailand; 2 Department of Prosthodontics, Faculty of Dentistry, Chulalongkorn University, Bangkok, Thailand

**Keywords:** zirconia, silicon carbide, mechanical polishing, polishing paste

## Abstract

**Objective:**

The aim of this study was to newly develop a silicon carbide polishing paste that was comparable to or more effective than diamond polishing paste for the final polishing step of zirconia.

**Materials and Methods:**

Fifty-two zirconia specimens were prepared, and polished with silicon carbide sandpaper to generate initial surface roughness. The surface roughness at baseline (Ra value) was measured by a profilometer and the specimens were randomly divided into six groups, which the first group ( *n* = 2) was used to study the surface morphology at baseline. The second to fifth groups ( *n* = 10/group) were polished for 30 seconds with different ratios of silicon carbide paste; silicon carbide:glycerin by weight: 1:1 (SiC1), 1.5:1 (SiC1.5), 2:1 (SiC2), and 2.5:1 (SiC2.5) according to their groups. The sixth group ( *n* = 10) was polished for 30 seconds with diamond paste (Dia). Afterward, the Ra values were remeasured at every 30-second polishing interval up to a total polishing time of 120 seconds. Scanning electron microscopy (SEM) was used to examine the surface morphology of postpolished specimens and the abrasive particles.

**Statistical Analysis:**

The differences in mean Ra values were analyzed using two-way repeated analysis of variance followed by least significant difference post hoc analysis. All tests were conducted at a significance level of 5% ( *p* < 0.05).

**Results:**

Within each group, the mean Ra values significantly decreased with longer polishing time ( *p* < 0.05), except the SiC2.5 group at 120 seconds. Increasing silicon carbide concentration significantly decreased the Ra values ( *p* < 0.05), with the exception of the SiC2.5 group. After 120 seconds, the SiC2 group demonstrated the lowest mean Ra value. The surface images investigated by SEM corresponded with their Ra values.

**Conclusion:**

Polishing zirconia with a silicon carbide paste, silicon carbide:glycerin ratio of 2:1 by weight, for 120 seconds, yields the smoothest postpolished surface. Furthermore, the mean Ra value obtained with this paste is statistically comparable to that of the diamond paste. Thus, silicon carbide paste has the potential to be an efficient alternative to diamond paste for chairside polishing of zirconia.

## Introduction

In restorative dentistry, dentists commonly adjust the glazed surfaces of zirconia during the try-in procedure. This creates surface roughness (Ra), which results in plaque accumulation, gingival inflammation, wear of the opposing dentition, and reduction of the strength and esthetics of the restorations. 1 2 3 Previous studies indicated a relationship between Ra value and bacterial adhesion, concluding that an Ra value above 0.2 μm increases the likelihood of bacterial attachment compared to smoother surfaces. 4 5 Therefore, polishing and/or reglazing after adjustment are necessary to restore zirconia's smooth surface. While reglazing requires additional visits, polishing can be done in a single visit, which is more convenient. 2

Polishing dental ceramic requires sequential steps. Coarse finishing burs (100–500 μm) are initially used to contour the bulk of the material, followed by fine, superfine burs, rubber abrasive points, and/or fine-particle discs (8–20 μm), respectively, to eliminate deep scratches and smoothen the surface. The final step involves the use of polishing pastes to generate an enamel-like gloss appearance of the restoration. 6 7 8 9

Diamond paste is considered the most effective polishing paste among abrasive materials. 2 10 As the hardest and most incompressible substance, 11 diamond is the standard material for polishing ceramic restorations. 12 13 Another commonly used abrasive is silicon carbide (SiC), whose hardness is second only to diamond. In dentistry, SiC is typically used in polishing burs, including rubber tips, coated disks, and brush-like burs. 14

Zirconia polishing is challenging due to its exceptional mechanical properties, leading to growing interest in identifying the most effective polishing protocols. 15 Furthermore, the use of polishing pastes is particularly crucial for smoothing pits and fissures of the restorations, which are inaccessible to polishing burs. 6 In addition, to the best of the authors' knowledge, SiC has only been used in polishing burs, with no SiC polishing paste currently available in the market.

The aim of the present study was to develop a novel silicon carbide polishing paste that is comparable to, or more effective than, diamond polishing paste for the final polishing step of zirconia. The null hypothesis was that surface roughness (Ra) of zirconia polished with silicon carbide polishing paste would not be significantly different from that of zirconia polished with diamond polishing paste.

## Materials and Methods

### Sample Size Calculation

The sample size of this study was calculated using mean Ra values and standard deviations which were obtained from the pilot study. The sample size calculation was performed with G*Power software (G*Power 3.1, Heinrich-Heine-Universitat Dusseldorf). The minimum sample size was 8 specimens per group. However, regarding compensating for the 20% error, 10 specimens per group were selected. Thus, including two unpolished specimens, a total of 52 specimens were required.

### Specimen Preparation

Fifty-two monochromatic zirconia specimens were prepared from presintered zirconia discs (Cercon xt shade A1, Dentsply Sirona, Erlangen, Germany) by cutting them into cuboidal shapes (7 mm in length, 6 mm in width, and 4 mm in thickness) using a low-speed precision cutting machine (IsoMet 1000 No. 11-2180, Buehler, Lake Bluff, Illinois, United States). The specimens were ultrasonically cleaned with distilled water for 5 minutes (CP360 Powersonic, Crest Ultrasonics, Ewing Township, New Jersey, United States), rinsed with distilled water and dried. All of the specimens were fully sintered in a furnace (Infire HTC Speed, Dentsply Sirona, Charlotte, North Carolina, United States) according to the manufacturer's instructions. After sintering, they were cooled down in the furnace.

**Fig. 1:**
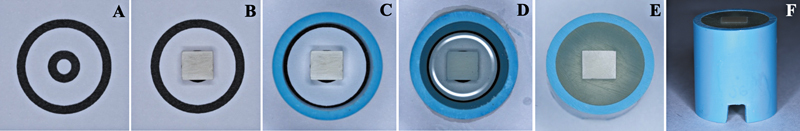
Specimen preparation. ( **A** ) A template was used to determine the position of the specimen (inner circle), and the polyvinyl chloride pipe (outer circle). ( **B** ) The specimen was attached to the template at the center position. ( **C** ) The pipe was secured over the specimen. ( **D** ) The specimen was embedded in epoxy resin. ( **E** ) The specimen was detached from the template. ( **F** ) A registration mark was created.

A plastic template was used to locate the position of each specimen and the polyvinyl chloride pipe. The specimen and the pipe were attached to the template with adhesive tape at the center and border positions, respectively. Consequently, epoxy resin was poured into the pipe. After the epoxy resin had completely hardened, a registration mark (4 mm in height and 6 mm in width) was made at the bottom of each pipe, which was used to align the specimen in the same position during surface roughness measurements ( Fig. 1 ).

For simulation of initial surface roughness, 6 specimens/round were polished for 10 minutes with 80-grit SiC sandpaper (3M Wetordry abrasive sheet, 3M, St. Paul, Minnesota, United States) by a polishing machine with an automatic head (NANO 2000 grinder-polisher with FEMTO-1000 polishing head, Pace Technologies, Tucson, Arizona, United States). The sandpaper was rotated at 200 revolutions per minute (rpm) clockwise, and the specimens were rotated at 200 rpm counterclockwise. The applied pressure was 1 kg/cm ^2^ , and a new sandpaper was replaced after each polishing cycle. Then, the specimens were ultrasonically cleaned in distilled water for 5 minutes, rinsed with distilled water, and dried.

### Baseline Surface Roughness Measurement

The surface roughness measurement (Ra value) at baseline was performed using a profilometer (Talyscan 150, Taylor Hobson, Leicester, United Kingdom). Five 2-mm measurement streaks were taken at the center of each specimen (with a cutoff value of 0.25 mm, and a stylus speed of 0.5 mm/s). The vertical distance between transverse measurement streaks was 0.4 mm. After the measurement, the specimen was rotated 90 degrees counterclockwise, and the Ra was measured using the same procedure. All specimens were aligned in the same position following the registration mark. After that, the mean Ra value of each specimen was calculated.

### Specimens Grouping

The specimens were randomly divided into six groups. The first group ( *n* = 2) was used to study the surface morphology at baseline. The second to fifth groups ( *n* = 10/group) were polished with various concentrations of SiC pastes. The sixth group ( *n* = 10), which was the control group, was polished with diamond paste (Dia).

### Polishing Paste Preparation

The SiC polishing pastes consisted of glycerin (99.5% glycerol, Qrec, New Zealand) as a lubricant and SiC particles (SiC abrasive powder, 0.1–1 μm diameter particles, Aldrich, Sigma-Aldrich Pte Ltd, Singapore) as abrasive particles. Diamond paste (Ultradent Diamond Polish Mint, 1 μm diamond particles in a water-soluble gel base, Ultradent Products, Utah, United States) was used as a polishing agent in the control group. The silicon carbide pastes were prepared in the different ratios of SiC:glycerin by weight: 1:1 (SiC1), 1.5:1 (SiC1.5), 2:1 (SiC2), and 2.5:1 (SiC2.5). The pastes were prepared by weighing the components to within 0.0001 g using an analytical balance (Precisa 40SM-200A, Precisa Gravimetrics AG, Dietikon, Switzerland), based on Table 1 . All polishing pastes were mixed with a spatula for 5 minutes and the mixtures were loaded into a 0.1- to 1-mL syringe (Slip-tip disposable tuberculin syringe, Medline Industries, Northfield, Illinois, United States). The polishing pastes were stored at room temperature (25°C) and had to be used within 12 hours.

**Table 1:** The compositions of polishing pastes

Group	Polishing paste composition (by weight)
SiC1	Silicon carbide (1.0): Glycerin (1.0)
SiC1.5	Silicon carbide (1.5): Glycerin (1.0)
SiC2	Silicon carbide (2.0): Glycerin (1.0)
SiC2.5	Silicon carbide (2.5): Glycerin (1.0)
Dia	Diamond paste (control group)

### Polishing Method

Note that 0.1 mL of each polishing paste was ejected onto the specimens. All specimens were polished in the same direction for 30 seconds using a felt polishing wheel (2.2 mm diameter, Felt Wheel, Jota) that was mounted onto a micromotor unit (K4-Knee control unit type 4964, KaVo, Biberach, Germany). A new polisher was changed for each specimen. The revolution speed was 6,000 rpm, and the polishing pressure was 40 g representing light pressure. All polishing procedures were performed by a single operator who was intracalibrated prior to, and during the polishing procedure every 10 specimens using a precision scale (METTLER TOLEDO Advanced MR Precision Balance, Mettler Toledo, Columbus, Ohio, United States). 16 After that, the specimens were ultrasonically cleaned in distilled water for 5 minutes, rinsed with distilled water, and dried.

### Repetitive Surface Roughness Measurement

Ra measurements of the specimens were taken after 30 seconds of polishing. The measurements were performed in the same manner as the baseline roughness measurement. After that, the specimens were ultrasonically cleaned in distilled water for 5 minutes, rinsed with distilled water, and dried.

An additional 30 seconds of polishing was repeated until 120 seconds—that is, 30, 60, 90, and 120 seconds of polishing—and the Ra values of the specimens were remeasured at every 30-second interval.

### Scanning Electron Microscopy Analysis

Two postpolished specimens from each group at 120 seconds and two unpolished specimens (baseline roughness) were removed from the epoxy resin, ultrasonically cleaned in distilled water for 5 minutes, rinsed with distilled water, and dried. The specimens were mounted onto adhesive-coated aluminum stubs (1 sample/stub), and gold sputter-coated (100 seconds, 50 mA) using a sputtering device (JFC-1200 Fine Coater, JEOL, Tokyo, Japan). The surface images were captured using a scanning electron microscopy (SEM) (QuantaTM 250 FEG scanning electron microscope, FEI, Hillsboro, Oregon, United States) with a 20-kV accelerating voltage at 350× and 500× magnifications.

SiC and diamond particles were obtained by smearing the SiC abrasives and diamond paste onto a glass slide, then rinsing with pure ethanol, and drying. Each glass slide was mounted onto an adhesive-coated aluminum stub, and sputter-coated with platinum (100 seconds, 50 mA) using the same sputtering device. Their morphology was examined using an ultrahigh-resolution field emission SEM (JSM-IT800 Schottky, JEOL). The analysis was conducted at an accelerating voltage of 5.0 to 10.0 kV, with magnifications of 20,000× and 30,000× for SiC and diamond particles, respectively.

### Statistical Analysis

The data were statistically analyzed using IBM SPSS statistics software version 29 for Windows (IBM Corporation, Armonk, New York, United States). The normality of the data distribution and the homogeneity of variance were analyzed using the Shapiro–Wilk test and Levene's test, respectively. The differences in mean Ra values between groups were analyzed using two-way repeated analysis of variance (ANOVA) followed by least significant difference post hoc analysis at a 95% significance level ( *p* < 0.05).

## Results

Two-way repeated measures ANOVA revealed significant effects of both groups ( *p* < 0.001), and times ( *p* < 0.001) on surface roughness, as well as a significant interaction between these two factors ( *p* < 0.001) ( Table 2 ).

**Table 2:** Effect of groups, times, and interaction on mean Ra values

Source	Sum of squares	*df*	Mean squares	*F*	*p*
Groups	0.002	4	0.000	10.456	< 0.001
Times	0.05	2.733	0.018	1859.884	< 0.001
Groups*Times	0.001	10.932	4.883E-05	4.960	< 0.001

Note: *F* is a ratio of the variance between groups to the variance within groups.

Abbreviation: df, degrees of freedom.

**Fig. 2:**
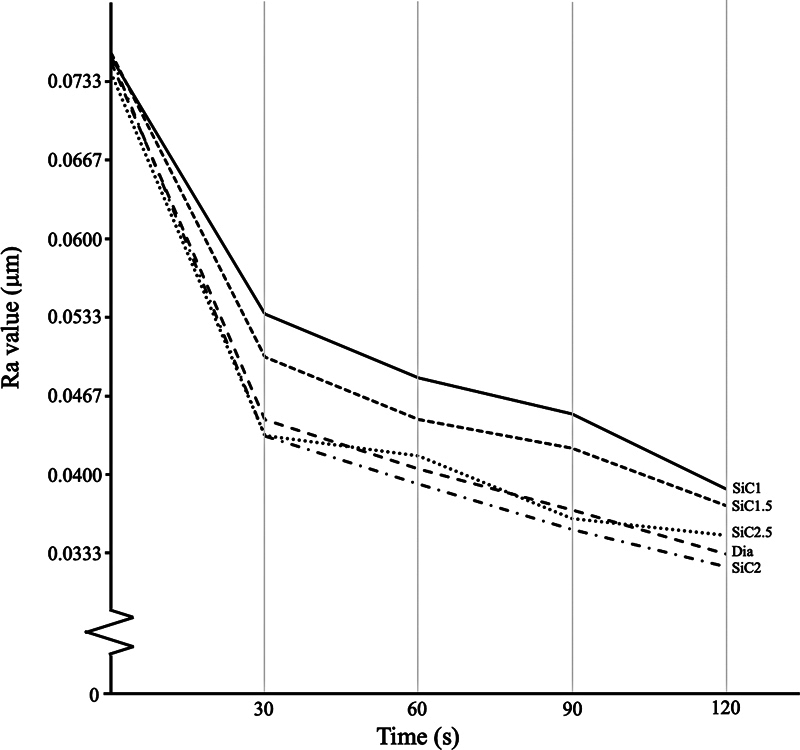
The mean Ra values of the groups at each time point (before and after polishing for 30, 60, 90, and 120 seconds).

The mean Ra values, standard deviations, and significant differences between groups and times are remarked in Table 3 . At baseline, mean Ra values were not significantly different between the groups ( *p* ≥ 0.05). Within each group, the mean Ra values significantly decreased as the polishing time increased ( *p* < 0.05), except for the SiC2.5 group at 120 seconds ( Table 3 , Fig. 2 ). The SiC2 group demonstrated the lowest mean Ra values, which were significantly lower than those of the SiC1 and SiC1.5 groups ( *p* < 0.05), but not significantly different from the SiC2.5 and Dia groups ( *p* ≥ 0.05). No significant differences in mean Ra values were observed between the SiC2, SiC2.5, and Dia groups across all polishing times ( *p* ≥ 0.05).

**Table 3:** Mean Ra values and standard deviation of the groups at baseline and after polishing for 30, 60, 90, and 120 seconds

Group	Ra (μm) ± SD				
	Baseline	30 s	60 s	90 s	120 s
SiC1	0.07534 ± 0.00264 ^A^	0.05360 ± 0.00292 ^B^	0.04818 ± 0.00243 ^D^	0.04512 ± 0.002160 ^G^	0.03877 ± 0.005960 ^I^
SiC1.5	0.07571 ± 0.00294 ^A^	0.04995 ± 0.00700 ^B^	0.04465 ± 0.00612 ^D,E^	0.04221 ± 0.00616 ^G^	0.03735 ± 0.00426 ^I,J^
SiC2	0.07537 ± 0.00266 ^A^	0.04324 ± 0.00599 ^C^	0.03917 ± 0.00480 ^F^	0.03531 ± 0.004112 ^H^	0.03216 ± 0.00414 ^K^
SiC2.5	0.07399 ± 0.00155 ^A^	0.04328 ± 0.00340 ^C^	0.04156 ± 0.00307 ^E,F^	0.03624 ± 0.00257 ^H,a^	0.03482 ± 0.00148 ^J,K,a^
Dia	0.07492 ± 0.00208 ^A^	0.04463 ± 0.00194 ^C^	0.04045 ± 0.00174 ^F^	0.03697 ± 0.00172 ^H^	0.03324 ± 0.00166 ^K^

Abbreviation: SD, standard deviation.

Note: Same superscript capital letter represents no significant difference between groups ( *p* ≥ 0.05) by two-way repeated analysis of variance (ANOVA) with least significant difference (LSD) post hoc analysis. Same superscript small letter represents no significant difference between times ( *p* ≥ 0.05) by two-way repeated ANOVA with LSD post hoc analysis.

**Fig. 3:**
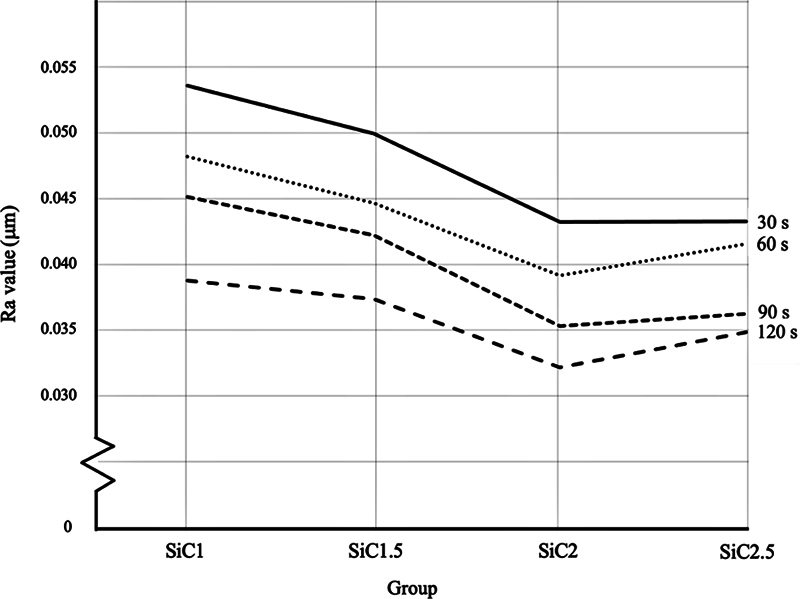
The relationship between the experimental groups polished with different ratios of silicon carbide pastes at each time point.

Fig. 3 illustrates the relationship between mean Ra values and SiC paste ratios across various polishing times. The line graphs demonstrate that the SiC2 group exhibited the lowest mean Ra value. In general, an increase in the SiC concentration resulted in a reduction in Ra values, with the exception of the SiC2.5 group, which did not follow this trend.

**Fig. 4:**
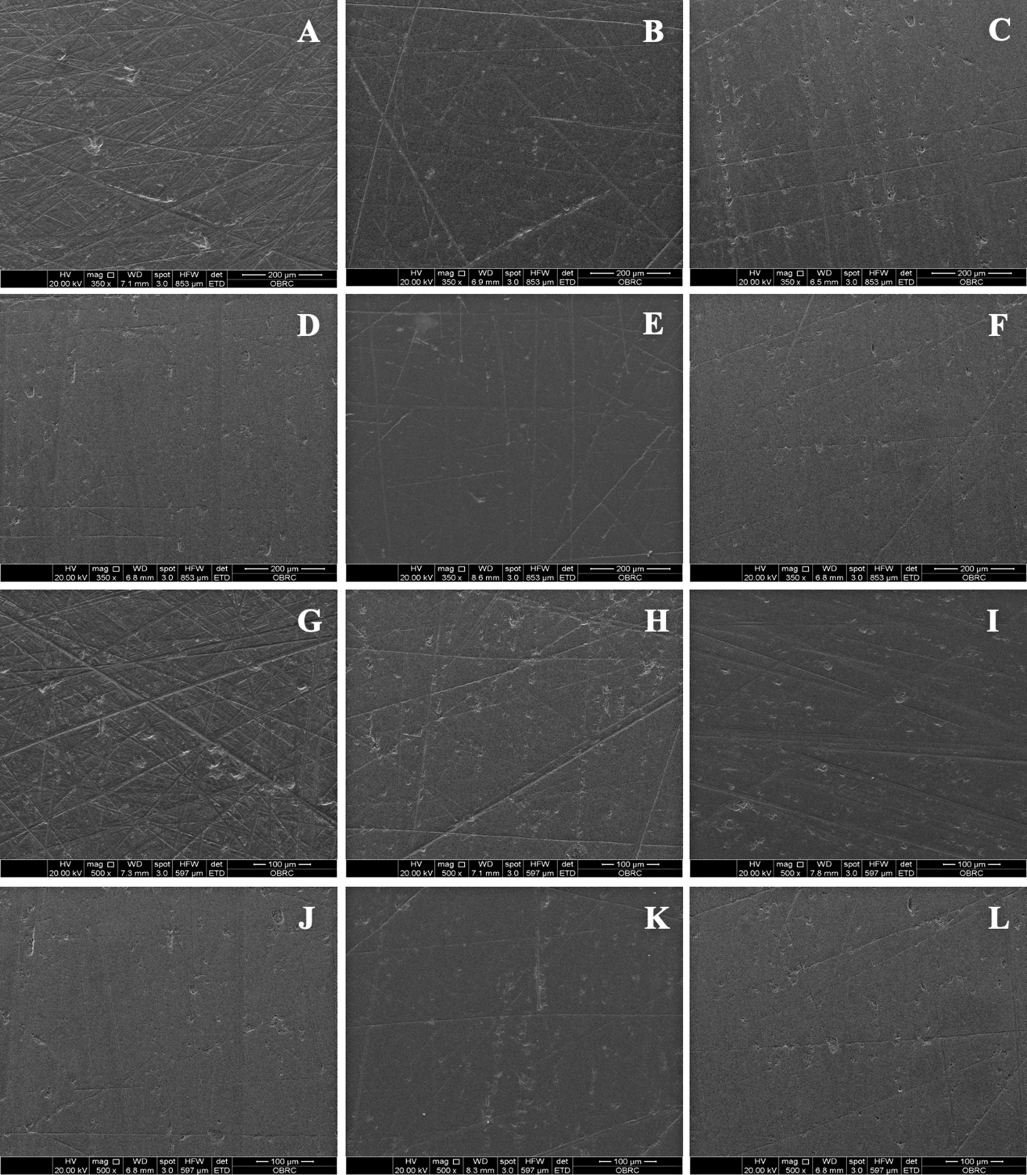
Scanning electron microscope (SEM) images at 350× ( **A** – **F** ) and 500× ( **G** – **L** ) magnifications of the zirconia polished with different polishing pastes. ( **A** ) Surface of zirconia before polishing (baseline), ( **B** ) SiC1, ( **C** ) SiC1.5, ( **D** ) SiC2, ( **E** ) SiC2.5, ( **F** ) Dia, ( **G** ) surface of zirconia before polishing (baseline), ( **H** ) SiC1, ( **I** ) SiC1.5, ( **J** ) SiC2, ( **K** ) SiC2.5, ( **L** ) Dia.

The surface images of the specimens, investigated under SEM at 350× and 500× magnifications ( Fig. 4 ), corresponded with their Ra values. At baseline, the specimens exhibited the roughest surfaces, whereas the SiC2 group demonstrated the smoothest surface among all groups ( Fig. 4 ).

**Fig. 5:**
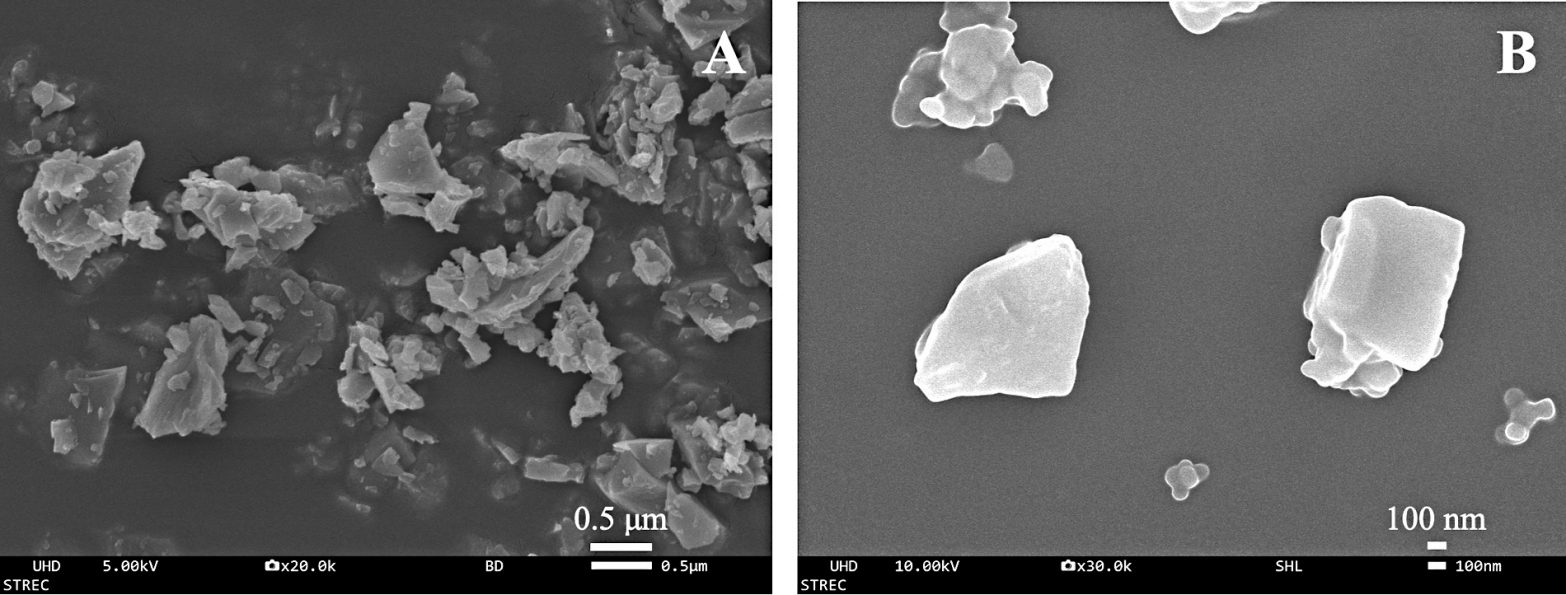
Scanning electron microscope (SEM) images of the polishing particles. ( **A** ) Silicon carbide particles at 20,000× magnification. ( **B** ) Diamond particles at 30,000× magnification.

SEM analysis of the abrasive particles revealed distinct morphological characteristics for each abrasive type. SiC particles were irregularly shaped with sharp cutting edges, while diamond particles exhibited irregularity in shape with rounder edges. Additionally, the diameter of the SiC particles ranged from 0.1 to 1 μm, whereas the diamond particles measured approximately 1 μm ( Fig. 5 ).

## Discussion

This study investigated the effectiveness of the SiC polishing pastes with different concentrations compared to diamond polishing paste for zirconia polishing. The results presented no significant difference in mean Ra values between the SiC2, SiC2.5, and Dia groups ( *p* ≥ 0.05). Therefore, the null hypothesis, which is “surface roughness (Ra) of zirconia polished with silicon carbide polishing paste would not be significantly different from that of zirconia polished with diamond polishing paste,” was accepted.

A profilometer commonly provides quantitative information about surface texture by generating Ra values. A high Ra value indicates a rough surface, while a low Ra value represents a smooth surface. A previous study recommended selecting parameters that both quantify the roughness and provide information on the morphology. 17 Whereas Ružbarský and Kukiattrakoon et al also suggested that noncontact profilometry is not suitable for measuring the roughness of shiny surfaces due to scattered light. 18 19 Thus, in this study, surface roughness was analyzed using both a tactile profilometer and SEM to examine the data quantitatively and qualitatively. Although it has been claimed that the stylus tip may cause surface damage to the specimen, 19 no abraded lines were observed in the SEM analysis of this study.

From the results ( Table 2 ), it was indicated that increasing both the concentration of SiC and polishing time resulted in a more effective polishing process than increasing either parameter individually. Within each group, mean Ra values significantly decreased over time ( *p* < 0.05), with the exception of the SiC2.5 group at 120 seconds ( Fig. 2 ). These findings align with those of Watanabe et al, who reported that longer polishing durations improve surface finish. 20 Furthermore, across the groups, the Ra values generally decreased with increasing SiC concentration. The SiC2 group consistently achieved the lowest mean Ra value ( Fig. 3 ). These results are in agreement with previous studies indicating that higher concentrations of polishing agents contribute to reduced surface roughness. 21 22

In this study, the mean Ra values decreased as the abrasive concentration increased. However, an increase in abrasive content from SiC2 to SiC2.5 led to a rise in mean Ra values ( Fig. 3 ). This phenomenon may be attributed to two possible explanations. First, as observed by Yamockul et al in 2016, high-viscosity pastes result in substantial polishing material loss during polishing, as the paste is prone to splattering, and fails to adhere effectively to the surface. 6 Second, in 2017, Alam et al proposed that at lower abrasive concentrations, there is sufficient space between the polisher and the substrate to accommodate all abrasive particles. This allows each abrasive particle to come in contact with the substrate and function effectively. However, when the abrasive concentration becomes too high, the particles are densely packed, reducing the number of abrasives that can actively engage with the surface. This overcrowding diminishes polishing effectiveness, resulting in increased mean Ra values, as observed in the SiC2.5 group. 23

SEM analysis of the postpolished specimens was consistent with their Ra values. The two unpolished specimens, which had the highest Ra values, displayed the roughest surface, characterized by deep scratches and large pits. In contrast, the SiC2 group, which possessed the lowest Ra value, exhibited the smoothest surface with only minimal abrasion lines and shallow cavities. All postpolishing groups presented smoother surfaces compared to the baseline ( Fig. 4 ).

Mechanical polishing is a conventional technique that primarily relies on abrasive agents to mechanically refine surface. 24 Material removal rate (MRR) of polishing pastes is influenced by several factors, including the relative surface hardness between the abrasive particles and the substrate, particle size and shape, polishing speed, polishing pressure, and the lubricants. 6 9 25 A previous study indicated that using abrasive particles harder than the substrate tends to cause scratches, but results in high MRR. 9 Therefore, SiC (Mohs hardness = 9.5), which is higher than zirconia (Mohs hardness = 8), was selected as an abrasive in this study. Particle size critically affects polishing efficiency; while larger particles abrade the surface faster, they tend to leave deeper scratches than smaller particles. 6 9 16 From this study, 0.1 to 1 μm SiC particles and 1 μm diamond particles were selected, with the SiC particles being notably finer. Furthermore, particle shape also plays an important role in polishing effectiveness. Particles with sharp cutting edges typically abrade surfaces more rapidly. 9 26 In this study, SiC particles, which have an irregular shape and sharp edges, and diamond particles with a comparatively rounded shape were used ( Fig. 5 ). Taken together, these factors suggest that SiC paste may serve as a more effective polishing agent than diamond paste.

Using SiC as an abrasive has an advantage over diamond. A study by Shih et al demonstrated that the comminution of SiC particles induces crack propagation on their surfaces, leading to particle fragmentation. 27 This fragmentation generates new cutting edges during the polishing process, thereby enhancing polishing efficiency. In contrast, such an effect is difficult to observe with diamond particles, as their superior surface hardness makes them highly resistant to breakage. 28 29 Another noteworthy consideration is the cost: commercially available SiC particles are dramatically less expensive than diamond particles, making SiC a promising, cost-effective alternative for polishing applications.

Lubricants are an important component of the polishing paste. Water-soluble lubricants are commonly used in the paste formulation because they are easier to clean than oil-based ones. 6 For this reason, glycerin was used as the lubricant in the present study. Regarding the type of polishing applicator, a recent study reported no significant differences between felt wheels, buff discs, or bristle brushes when polishing ceramic. 13 Furthermore, buff discs require a substantial amount of polishing paste, while bristle brushes tend to cause paste splattering. 6 As a result, felt wheels were selected as the polishing device in this study.

To achieve an enamel-liked surface finish, polishing paste is recommended to be used in the final step of polishing. 30 31 Thus, the authors suggested SiC polishing paste as an alternative paste when polishing zirconia. Moreover, the results of the present study can be applied in clinical practice. Clinicians can easily make the SiC polishing paste for chairside polishing by mixing the SiC particles with glycerin and loading it into a syringe. Since the SiC2 group showed the lowest mean Ra values, which were not significantly different from the SiC2.5 and Dia groups ( *p* ≥ 0.05), a SiC:glycerin ratio of 2:1 by weight is recommended. Moreover, a felt wheel is advised to be used as a polishing applicator.

This study has several potential limitations. First, as an *in vitro* investigation, the effectiveness of the polishing paste may not fully replicate clinical conditions. Additionally, variations in results may arise due to differences in specimen shapes, types, or brands of ceramics, and the polishing techniques used.

Further research is warranted to evaluate surface roughness in crown-shaped specimens, across various zirconia types, and using different polishing protocols. Moreover, the use of additional surface roughness parameters may be necessary to better capture changes in surface characteristics following polishing.

## Conclusion

Within the limitations, polishing zirconia with a 0.1- to 1-μm silicon carbide paste, with a silicon carbide:glycerin ratio of 2:1 by weight, for 120 seconds, yields the smoothest postpolished surface. Furthermore, the mean Ra value obtained with this paste is statistically comparable to that of the 1-μm diamond paste ( *p* ≥ 0.05). Thus, the SiC2 paste may serve as an effective and efficient alternative to diamond paste for chairside zirconia polishing in the final polishing step.
